# The Combined Effect of Neuropsychological and Neuropathological Deficits on Instrumental Activities of Daily Living in Older Adults: a Systematic Review

**DOI:** 10.1007/s11065-015-9312-y

**Published:** 2016-01-05

**Authors:** Eduard J. Overdorp, Roy P. C. Kessels, Jurgen A. Claassen, Joukje M. Oosterman

**Affiliations:** Department of Medical Psychology, Gelre Medical Centre, Zutphen, The Netherlands; Radboud University Nijmegen, Donders Institute for Brain, Cognition and Behaviour, Montessorilaan 3, 6500 HE Nijmegen, The Netherlands; Department of Medical Psychology, Radboud University Medical Center, Nijmegen, The Netherlands; Department of Geriatric Medicine and Radboud Alzheimer Center, Radboud University Medical Center, Nijmegen, The Netherlands

**Keywords:** Memory, Executive function, Magnetic resonance imaging, White matter, Gray matter, Hippocampus, Instrumental activities of daily living

## Abstract

To date, studies have consistently demonstrated associations between either neuropsychological deficits or neuroanatomical changes and instrumental activities of daily living (IADL) in aging. Only a limited number of studies have evaluated morphological brain changes and neuropsychological test performance concurrently in relation to IADL in this population. As a result, it remains largely unknown whether these factors independently predict functional outcome. The current systematic review intended to address this lack of information by reviewing the literature on older adults, incorporating studies that examined e.g., normal aging, but also stroke or dementia patients. A comprehensive search of databases (Pubmed, Embase, Medline, Web of Science, PsycINFO) and reference lists was performed, focusing on papers in the English language that examined the combined effect of neuropsychological and neuroanatomical factors on IADL in samples of adults with an average age above 50. In total, 58 potential articles were identified; 20 were included in the review. The results show that especially neuropsychological variables (primarily memory and executive functions) independently predict IADL. Although some unique predictive value of brain morphological changes, such as hippocampal atrophy, was found, support for the importance of white matter changes was limited. However, the results of the studies reviewed are diverse, and appear to be at least partially determined by the variables included. For example, studies were less likely to find an independent effect of cognition if they solely employed a cognitive screening instrument. This indicates that a structured examination of neuroanatomical and neuropsychological correlates of IADL in different patient populations is warranted.

**Electronic supplementary material:**

The online version of this article (doi:10.1007/s11065-015-9312-y) contains supplementary material, which is available to authorized users.

## Introduction

Activities of daily living can be divided into basic activities of daily living (ADL) and instrumental activities of daily living (IADL). ADL consist of activities of self-maintenance such as feeding, dressing and toileting (Lawton and Brody 1969). IADL include complex behaviours of domestic functioning, such as food preparation, financial administration, housekeeping, use of telephone, responsibility for own medication, mode of transportation and shopping. In older adults with cognitive decline, such as dementia or vascular cognitive impairment, daily-living skills are often compromised. Moreover, even though normal aging is by definition characterized by intact IADL, some studies have illustrated that a lower level of education and an advanced age are associated with poorer functional status (Artero et al. 2001), as are chronic disease burden and lifestyle factors (Wang et al. 2002). Deficits in IADL have been linked to increased distress and a reduced quality of life in patients and their caregivers as well as an increased use of healthcare services (Hope et al. 1998; Vetter et al. 1999). Given the significant burden of functional impairments on patients, caregivers, and care providers, identifying factors that best predict future decline in daily functioning has important clinical implications. ADL and IADL can be affected by conditions that disturb mobility (e.g. stroke, amputation, arthritis) or visual and auditory perception. In the current review, the focus is on two other important correlates of IADL: neuropsychological and morphological brain changes.

Individuals with Alzheimer’s disease (AD), one of the most studied clinical populations, are commonly characterized by impairments in IADL. In the initial phases of AD, independent accomplishment of IADL is altered. The progressive decline later affects ADL as well (Gauthier et al. 1997). IADL functions require a greater complexity of neuropsychological organisation than ADL functions, and are therefore more likely to be sensitive to the early effects of cognitive decline (Pérès et al. 2008; Wicklund et al. 2007). In contrast, ADL, including tasks such as grooming, toileting, and feeding, correlate strongly with motor functioning and coordination (Boyle et al. 2002; Cahn et al. 1998).

In clinical practice, IADL impairments by definition determine the difference between the mild cognitive impairment (MCI) and dementia stage of neurodegenerative diseases, such as Alzheimer’s disease. As indicated in the previous section, IADL variables and neuropsychological deficits are closely related, but other determinants of IADL have also recently been identified. For example, neuroanatomical changes may act as a common pathway of deficits in both neuropsychological test performance and IADL, explaining their interrelationship. Previous studies have indeed illustrated a relationship between IADL and neuroanatomical outcome measures, such as white matter hyperintensities (Inzitari et al. 2009). A crucial question therefore is whether neuroanatomical and neuropsychological correlates are independently related to IADL. Most studies to date have focused on either neuropsychological or neuroanatomical correlates of IADL in isolation. The goal of this review is to examine the extent to which neuropsychological and neuroanatomical correlates independently predict IADL. In the next sections, we provide a short summary of the neuropsychological and neuroanatomical factors that have been identified as the most important correlates of impaired IADL, followed by a review of those studies that examined neuroanatomical and neuropsychological correlates of IADL concurrently.

## The Relation between Neuropsychological Deficits and IADL

The first studies on this association showed that certain IADL activities are sensitive to early neuropsychological decline, and as such may be useful as predictors of future severe cognitive deterioration. Most earlier studies demonstrated a relation between global cognitive impairment, as assessed by screening instruments such as the Mini-Mental State Examination (MMSE; Folstein et al. 1975), and measures of functional status (Reed et al. 1989; Skurla et al. 1988). Later studies (see next sections) distinguished between different cognitive domains and examined these either in isolation or in combination to IADL. Most of these studies were cross-sectional in nature; a handful of studies also examined longitudinal relationships.

### Cross-Sectional Results

From a theoretical perspective, particularly executive functions may be relevant for IADL performance. These functions are generally defined as the abilities to perform complex, goal-directed, and self-serving behaviours (Brennan et al. 1997). Similarly, IADL are defined as complex, real-world adaptive human behaviours that require independence, volition, organizational ability, judgment and sequencing (Lawton 1988). Without appropriate executive control, individuals have difficulty appropriately initiating and completing IADL. Up until now, the majority of studies indeed point to strong associations between executive function and IADL. Previous work by Nadler et al. (1993) and Fogel and colleagues (Fogel 1994), for instance, demonstrated that measurements of executive functioning are reliable predictors of IADL in geriatric patients. More recent studies have confirmed these findings in other populations as well, including AD (Boyle et al. 2003a; Chen et al. 1998; Pereira et al. 2008; Razani et al. 2007; Senanarong et al. 2005; Stout et al. 2003), healthy community-dwelling elderly persons (Grigsby et al. 1998; Cahn-Weiner et al. 2000; Royall et al. 2004; Bell-McGinty et al. 2002), patients with Parkinson’s disease (Cahn et al. 1998) and elderly patients suffering from depression (Kiosses et al. 2000). These findings suggest that executive dysfunction may significantly affect the performance of complex activities that require goal-directed behaviour, organisation of an action and behavioural persistence.

In addition to executive dysfunction, a number of studies concluded that memory impairments are also associated with functional decline (Dunn et al. 1990; Goldstein et al. 1992; McCue et al. 1990; Richardson et al. 1995; Farias et al. 2004). Also studies that concurrently examined multiple cognitive domains, have demonstrated that memory and executive functioning are important correlates of IADL in AD (Farias et al. 2003; Matsuda and Saito 2005).

### Longitudinal Results

Most of the studies examining the relationship between cognition and functional impairment are cross-sectional in nature, and have not examined how memory and executive function at baseline predict the rate of future decline in IADL. Cross-sectional studies therefore provide only limited insight into the course of decline in cognition and IADL function. Understanding the patterns of change in these two domains provides a better explanation of the course of dementia. Many early longitudinal studies that evaluated cognitive functioning and its relation with future disability have relied on global measures of cognition, such as the MMSE. For example, population-based longitudinal studies have shown that global measures of lower baseline cognitive function are associated with faster rates of functional decline and that they predict the development of future disability in IADL (Barberger-Gateau and Fabrigoule 1997; Royall et al. 2004; Schmeidler et al. 1998). More recently, Farias et al. (2009) examined the association between longitudinal changes in domain-specific cognitive functions and the decline in IADL. They found that changes in both memory and executive functions were independently associated with the rate of decline in IADL.

## The Relation between Brain Morphological Changes and Instrumental Activities of Daily Living

Previous studies have revealed that neuropsychological functioning accounts for only a moderate proportion of variance in IADL (Reed et al. 1989; Nadler et al. 1993), indicating that other variables are important as well. For example, several studies have shown a significant association between IADL and white matter changes (WMC). Such findings have been reported in populations with predominantly subcortical ischemic vascular disease (SIVD) (Pohjasvaara et al. 2003, 2007) but also in patients with MCI (Yoon et al. 2013) or dementia (Moon et al. 2011; Park et al. 2011), as well as in community-dwelling older adults (Baune et al. 2009; Sonohara et al. 2008). A longitudinal study confirmed the important role of WMC in predicting future functional decline (Inzitari et al. 2009), by showing that older people with extensive age related changes in white matter are at a high risk of functional decline over the next three years.

In addition to WMC, some studies have highlighted the importance of gray matter atrophy in IADL (Yoshida et al. 2012). Hénon et al. (1998), for instance, found evidence that medial temporal lobe atrophy was associated with functional status in stroke patients. A recent longitudinal study demonstrated that parietal and temporal lobe atrophy at baseline predict worsening IADL impairment over time (Marshall et al. 2014). Nonetheless, up until now, most studies focused solely on WMC, and did not consider possible gray-matter alterations as important correlates of IADL.

## Interim Summary and Goal of the Present Review

Overall, the aforementioned studies suggest that particularly executive function is related to IADL. Similarly, most studies indicate that WMC are associated with IADL. The extent to which these observations are independent is, however, unclear. The strong association that has been reported between executive function and WMC (Schmidt et al. 2011) indicates that WMC may mediate the relationship between executive function and IADL. In the same line, both hippocampal atrophy and memory function have been identified as correlates of IADL, but the extent to which these relationships are independent of one another, considering their strong association (Kaup et al. 2011), is largely unknown. Only a limited number of studies have examined the predictive value of neuropsychological and morphological variables for IADL concurrently. The main goal of this review is therefore to describe the present knowledge of the interrelationships between, and the unique contributions of, cognitive functioning and MRI findings in relation to IADL in elderly populations. We hypothesized that the effects of brain morphological changes and neuropsychological test performance on IADL are independent. In addition, we hypothesized that the underlying clinical condition (e.g., AD) affects this relationship. Here, we expected that neurocognitive deficits characteristic for a specific disease would emerge as the most important correlate of IADL, for example memory and/or hippocampal atrophy for AD and executive function and/or WMC in diseases with vascular pathology.

## Methods

For this systematic review, an extensive literature search was conducted of online databases (Pubmed, Embase, Medline, Web of Science, and PsycINFO) until June 2015. The following inclusion criteria were employed: 1. The paper had to be published after 1988, based on the first population based studies concerning the relation between brain morphological changes, neuropsychological test performance and IADL in older adults; 2. The age of the study sample was restricted to a minimum of 50 years (on average), since from that age onwards significant changes in gray and white matter brain regions become apparent (Bartzokis et al. 2001; Walhovd et al. 2005); 3. The paper had to examine IADL in relation to neuropsychological and brain morphological changes concurrently. Only those articles explicitly addressing the question whether cognitive and/or neuropathological factors independently predict IADL in older adults were selected; 4. The paper had to be published in the English language; 5. The paper had to employ a validated measure of IADL, such as the Lawton & Brody IADL list, or had to refer to a previous study in which the list was validated; 6. Clinical diagnoses had to be made according to internationally-established guidelines.

Searches were conducted using (any truncated versions of) combinations of a keyword from each category: Category 1: Cognition (cognitive, cognition, memory, executive, visuospatial); Category 2: Imaging (MRI, magnetic resonance imaging, white matter, grey/gray matter, hippocampus); Category 3: IADL (activities of daily living, independence, functional impairment, functional decline, functional status, instrumental activities of daily living, IADL, ADL and vitality). IADL included the ability to perform the following tasks independently: food preparation, financial administration, housekeeping, use of telephone, responsibility for own medication, mode of transportation and shopping. The reference lists of all articles related to the study were screened, to identify additional relevant articles. Finally, in order to reduce the effect of a publication bias, several key authors of studies included in the review were contacted to inquire about the existence of unpublished study results on this topic.

EJO performed all initial searches and screened all abstracts of all articles that resulted from this search to identify potentially suitable papers; further selection of papers of interest for this study was conducted by EJO and JMO independently, and findings were discussed in a consensus meeting.

## Quality Assessment

The methodological quality of all studies was rated using the Newcastle-Ottawa Quality Assessment Scale. This scale measures three types of outcomes for which a total of 9 stars can be given. These are the Selection criteria (4 stars maximum), the Comparability criteria (2 stars maximum), and the Outcome criteria (3 stars maximum). Criteria were adjusted such that the use of volumetric or validated visual rating scales was necessary to assess brain morphological changes, together with the use of standardized and validated instruments to measure neuropsychological test performance. Groups had to be matched with regard to age (first Comparability criterion), and educational attainment/intelligence estimate or sex (second Comparability criterion), or these factors had to be statistically controlled for in case of cross-sectional data, as they present important potential confounds of IADL. Since the studies included in this review examined cross-sectional/correlational data to answer the research question of whether MRI variables and neuropsychological scores independently predict IADL within specified study samples, also in longitudinal studies, the criteria were adjusted so that they covered quality ratings of these kinds of correlational studies (see Herzog et al. 2013). Both EJO and JMO independently rated these criteria, and consensus was reached through discussions.

## Results

Based on the first screening of the titles and abstracts, the initial search resulted in 175 potential articles that were fully screened in order to identify potential studies for inclusion in this review, of which 58 explicitly referred to IADL in relation to cognitive and neuropathological deficits. A careful review of their reference lists did not identify any additional articles. A further detailed examination revealed that only 20 of these 58 studies explicitly examined the combined impact of cognitive and neuroanatomical factors on IADL, and met all specified inclusion criteria (Table 1). These studies focused on the following clinical populations: non-demented elderly people (*n* = 3), depressed elderly (depressed only: *n* = 2, depressed + non-depressed elderly: *n* = 2), vascular dementia patients (*n* = 3), MCI patients and elderly controls (*n* = 1), early AD and elderly controls (*n* = 2), elderly controls, MCI and dementia patients (*n* = 3), diabetic patients and elderly controls (*n* = 1), stroke patients with elderly controls (*n* = 1), MCI patients only (*n* = 1), and AD patients with cerebrovascular disease (*n* = 1). The authors that were contacted were not aware of unpublished study results; one unpublished dissertation was found, however, further inspection revealed that results from this dissertation were used as part of an already included article.

**Table 1:** Studies included in the review

Authors sample characteristics	Functional scale	Cross sectional or longitudinal	Neuropsychological tests	Neuroanatomical correlates	Results
Bennett et al. 2002. 77 subcortical VaD patients (age 73.42, range 60–68)	IADL subscale of Lawton and Brody *Informant-report*	Longitudinal, 6 years follow-up	MMSE/CDR/FAS/DF + DB + MC of WMS/Sum of 5 trails and trail 6 as a percentage of Trail 5 on RAVLT/Similarities and BD of WAIS-R/Percentage delayed recall of LM and VR of WMS/PM	CA VE NI Vol I LA *All visual*, *except VI which was volumetric*	DF and RAVLT trail 6 performance independently predicted IADL decline. No independent effect of neuroanatomical correlates was found.
Bennett et al. 2006. 106 Community-living nondemented individuals (age 85.2 ± 2.9)	The Kilsyth Disability Rating Scale, and an interview to measure complex IADL *Informant- and self-report*	Cross sectional	MMSE/DF + DB of WMS-R/BNT/SVFT/Oral TMT-B/LM + VR of WMS-R/Similarities of WAIS-R/Line Orientation Test/Copy of the cube drawing task/CES-D	PWML DWML HC Vol WBVol *PWML* & *DWML visual*, *rest volumetric*	DB and LM performance were related to cooking; Oral TMT-B with housework, and DF with shopping. DB and Similarities were related to Reading. Smaller left hippocampal volume was associated with less socializing (complex IADL). Uniqueness is assumed since no overlap is present between the neuroanatomical and neuropsychological correlates regarding individual IADLs.
Boyle et al. 2003b. 29 VaD patients (age 78.0 ± 5.66)	IADL subscale of Lawton and Brody *Informant-report*	Cross sectional	All subscales and total score of MDRS	SH CVol *Volumetric*	The I/P subscale of MDRS and SH independently contributed to the variance in IADLs. I/P alone accounted for 28 %, and SH accounted for 14 % beyond the contribution made by I/P.
Boyle et al. 2004. 28 VAD patients (age 78.0 ± 5.7)	IADL subscale of Lawton and Brody *Informant-report*	Longitudinal, 1 year follow-up	I/P subscale of MDRS/TMT-B/SCWT/MMSE	SH *Volumetric*	Baseline performance on executive tasks (MDRS I/P, SCWT) was a significant predictor of IADLs at the 1 year follow-up, explaining 50 % of the variance, even after accounting for global cognitive dysfunction (MMSE). SH did not explain unique variance in IADLs after accounting for executive dysfunction.
Brown et al. 2011. N = 816 394 amnestic-MCI (age 74.84 ± 7.40) 193 AD patients (age 75.33 ± 7.48) 229 controls (age 75.90 ± 5.00)	Pfeffer Functional Activity Questionnaire (FAQ) *Informant-report for aMCI* & *AD*, *self-report for controls*	Cross sectional	TMT-A + B/DSym of WAIS-R/AVLT	HC Vol IBVol ECVol *Volumetric*	Within the aMCI group, total severity of IADL impairment and the total number of deficits was independently associated with hippocampal volume, AVLT 30-min delay and TMT-A scores; DSym was additionally associated with total severity score. In the combined aMCI and AD group, hippocampal volume and Trail Making Test, part A score independently predicted total and average severity of IADL impairment, as well as the number of deficits. In addition, AVLT 30-min delay score and entorhinal cortex volume independently predicted total severity and total number of deficits. These relations were not examined in the control group.
Cahn et al. 1996. 30 elderly inpatients with major depression (age 75.5 ± 6.6)	A modified version of the Lawton & Brody IADL and Physical Self-Maintenance Scale. Total ADL score (PADL + IADL) was used *Informant-report*	Cross sectional	Immediate and delayed recall from the VR of WMS-R/Short and long delayed free recall of CVLT/SDMT/TMT-B/BNT/BCOWART/HVOT/RCFT/BCD/WCST/LGP	PVH DWMH WBA *Volumetric*	Neuropsychological test performance (NP Index, combining all tests), age, depression severity, and WMH (combined PVH & DWMH score) accounted for 53 % of the variance in ADL functioning. Severity of WMH accounted for an additional 18 % of the variance over and above the other 3 variables. The contribution of the NP Index was not significant.
Cahn-Weiner et al. 2007. N = 124 52 controls (age 72.5 ± 7.4) 35 MCI (age 72.8 ± 8.4) 37 Dementia (age 73.1 ± 8.4)	Blessed Roth Dementia Rating Scale (BRDRS). Only the eight IADLs were analysed *Informant-report*	Cross sectional & longitudinal, 5 years follow-up	The memory composite (MEM): the MAS Word List Learning Test The executive composite score (EXEC): DB of WMS-R/VMSB of WMS-R/I/P subscale of MDRS/Letter Fluency/FAS	LAC WMH CGMVol HC Vol *Volumetric*	In the entire sample, baseline MEM and hippocampal volume were independently associated with baseline IADL scores. Only baseline EXEC independently predicted rate of decline in IADL scores.
Chen et al. 2015. 66 AD patients with CVD (age 70.8 ± 10.7)	IADL scale of Lawton and Brody *Informant- report at follow-up*	Longitudinal, 6 months follow-up	MMSE	WML MTA VBR *WML* & *MTA visual*, *VBR volumetric*	WML and MMSE significantly predicted IADL both at baseline and at follow-up.
Christman et al. 2010. N = 178 28 diabetics (age 66.39 ± 12.54) 150 non diabetics (age 63.43 ± 13.29)	IADL scale of Lawton and Brody *Informant- and self-report*	Cross sectional	MMSE/DSym + DS + BD + PC of WAIS-R/BTA/Speed test of SPCT/TMT-A + B/letter-, category-, design fluency/Information and Similarities of WAIS-R/IQ portion of NART/Copy of RCFT/BFRT/Learning trails (1–3) + delayed recall trail of HVLT-R/LM I + II of WMS-R/Learning trails (1–3) + delayed recall trail of BVMT-R/VR I + II of WMS-R/WCST-m	B-to-ICVol *Volumetric*	In the diabetic group, lower B-to-ICV ratio was not associated with IADLs. In this group, only working memory and speed were associated with IADL; since no association with B-to-ICV was found, some uniqueness can be assumed. In the non-diabetic group no significant associations were found between cognitive domains, B-to-ICV and functional activities.
Griffith et al. 2010. N = 66 38 amnestic MCI (age 70.8 ± 6.4) 28 controls (age 71.6 ± 5.4)	Financial Capacity Instrument (FCI) *Performance-based*	Cross sectional	Spatial Span subtest of WMS-III/TMT-A/WRAT-3 Arithmetic subtest/MMSE	MFLVol AGVol HC Vol Pre. Vol *Volumetric*	In the amnestic MCI group, angular gyrus volume predicted the FCI scores after controlling for the MMSE. Separate mediation models indicated that Spatial Span and arithmetic ability partially mediated the relation between angular gyrus volume and FCI score. The effect of angular gyrus was marginally significant after controlling for Spatial Span; it was no longer significant controlling for arithmetic ability. These relations were not examined in the control group.
Hybels et al. 2014. N = 377 237 depressed patients (age 69.5 ± 7.3) 140 controls (age 70.4 ± 6.3)	The Duke Depression Evaluation Schedule, IADL examined separately from BADL and mobility *Self-report*	Cross sectional	MMSE	WMLVol *Volumetric*	MMSE, but not WML volume, predicted IADL impairment in the entire sample. Also in the subsamples, WML volume did not predict IADL impairment, whereas the MMSE did.
Inzitari et al. 2007. 619 elderly participants (age 74 ± 5)	IADL scale of Lawton and Brody *Informant-report*	Longitudinal, 1 year follow-up	ADAS-cognitive subscale	ARWMC *Visual*	The severity of ARWMCs significantly and independently predicts functional declining in a period of 1 year. Cognitive performance at baseline (ADAS-Cog) was an important factor underlying the effect of ARWMCs, but it is unclear whether cognition was also an independent significant predictor of IADL.
Kochan et al. 2011. 30 MCI patients (age 78.3 ± 4.0)	Bayer Activities of Daily Living *Informant-based*	Longitudinal, 2 year follow-up	MMSE	HC Vol GMVol PMCVol *Volumetric*	No independent effects of MMSE or neuroimaging variables were found.
Marshall et al. 2014. N = 812 229 controls (age 76.0 ± 5.0) 395 MCI (age 74.8 ± 7.5) 188 AD (age 75.3 ± 7.5)	Functional Activities Questionnaire (FAQ) *Self-report*	Cross sectional & longitudinal, 3 years follow-up	NART/RAVLT/DSym	Cortical thickness of: inferior temporal, supramarginal, precuneus, rostral anterior cingulate, medial orbitofrontal, rostral middle frontal, and lateral occipital cortices *Volumetric*	At baseline, worse DSym performance overall and decreased inferior temporal cortical thickness, increased lateral occipital cortical thickness in particularly AD patients predicted worse IADL. The unexpected effect of occipital thickness is likely due to multicollinearity. Lower baseline supramarginal cortical thickness and lower baseline inferior temporal cortical thickness predicted increased IADL decline. Additional linear level effects for precuneus cortical thickness and RAVLT were noted.
Mok et al. 2004. N = 117 75 SSVD patients (age 71.0 ± 11.2) 42 controls (age 69.6 ± 9.9)	IADL subscale of Lawton and Brody *Self-report*	Cross sectional	ADAS-cognition/I/P subscale of MDRS/CDR/MMSE	NSSI SRSI WMC *Visual*	Stroke severity and executive dysfunction (MDRS I/P), but not neuroimaging, contribute independently to poor IADL. These analyses were not conducted in the control group.
Steffens et al. 2002. 224 depressed adults (age > 60)	The Duke Depression Evaluation Schedule, IADL and BADL examined separately *Self-report*	Cross sectional	MMSE	WMLVol GMLVol *Volumetric*	MMSE independently predicted IADL, whereas WMLV and GMLV did not.
Stoeckel et al. 2013. N = 32 16 AD patients (age 76.94 ± 8.16) 16 controls (age 75.0 ± 4.43)	Financial Capacity Instrument *Performance-based*	Cross sectional	MMSE, DRS-2 (dividing between attention, initiation/perseveration, construction, conceptualization, memory)	MFCVol Pre Vol DFCVol AGVol HC Vol *Volumetric*	DRS-2 Attention and Construction scores mediate the effect of MFCV on IADL. These analyses were not conducted for the control group.
Taylor et al. 2003. N = 81 41 depressed elderly (age 68.73 ± 6.98) 40 controls (age 71.42 ± 6.07)	The Duke Depression Evaluation Schedule, IADL examined separately from BADL *Self-report*	Cross sectional	MMSE	TBVol TOFCVol *Volumetric*	In the entire sample, smaller OFC volumes and being depressed were significantly associated with IADL impairment. No unique contribution of MMSE scores was found.
Verlinden et al. 2014. 2025 stroke-free nondemented adults (age 59.9 ± 7.0)	IADL scale of Lawton and Brody *Self-report*	Cross sectional & Longitudinal, mean 5,7 years follow-up	MMSE	BVol GMVol TWMVol HC Vol WMLVol LI Microbleeds FA Mean-; axial-; radial diffusivity *Volumetric*	After controlling for MMSE, smaller brain volume and smaller hippocampal volume was associated with larger deterioration in IADL.
Vidoni et al. 2010. N = 114 56 controls (age 73.0 ± 6.2) 58 early AD (age 74.2 ± 6.3)	Alzheimer Disease Cooperative Study Activities of Daily Living Scale for Mild Cognitive Impairment (ADCS-ADL) *Informant-report*	Cross sectional	Global cognitive battery (COG) consisted of: LM IA + IIA of WMS-R/Free and Cued SRT/15 items of BNT/Letter - number sequencing of WAIS/DF + DB of WMSIII/TMT-A + B/SCWT/Verbal Fluency/BD of WAIS/MMSE	RGMA *Volumetric*	In demented subjects, reduced medial frontal cortex gray matter volume was a common substrate of both IADL and COG performance; in the non-demented subjects, the middle frontal and precentral cortex was the common substrate of IADL and COG performance.

**Abbreviations of neuropsychological tests:** ADAS, Alzheimer’s Disease Assessment Scale; AVLT, Auditory Verbal Learning Test; BCOWAT, Benton Controlled Oral Word Association Test (word fluency); BCD, Boston Clock Drawings; BD, Blok Design; BFRT, Benton Facial Recognition Test; BNT, Boston Naming Test; BTA, Brief Test of Attention; BVMT-R, Brief Visuospatial Memory Test-Revised; CDR, Clinical Dementia Rating Scale; CDT, Clock Drawing Test; CES-D, Center for Epidemiological Studies Depression Scale; CVLT, California Verbal Learning Test; DB, Digit Backward; DF, Digit Forward; DS, Digit Span; DSym, Digit Symbol; FAS, Phonemic Verbal Fluency; GDS, Global Deterioration Scale; GDS(−15), Geriatric Depression Scale (15 items); HVLT-R, Hopkins Verbal Learning Test-Revised; HVOT, Hooper Visual Organisation Test; IP, Initiation/Perseveration; LGP, Lafayette Grooved Pegboard; LM, Logical Memory; MC, Mental Control; MDRS, Mattis Dementia Rating Scale; MMSE, Mini Mental State Examination; NART, National Adult Reading Test; PM, Porteus Mazes; RAVLT, Rey Auditory Verbal Learning Test; RCFT, Rey-Osterrieth Complex Figure Test; SCWT, Stroop Color Word Test; SDMT, Symbol Digit Modalities Test; SENAS, Spanish and English Neuropsychological Assessment Scales; SHAS, Stanford Health Assessment Questionnaire; SPCT, Salthouse Perceptual Comparison Test; SPPB, Short Physical Performance Battery; SRT, Selective Reminding Test; SVFT, Semantic Verbal Fluency Test; TMT-A + B, Trail Making Test part A + B; VADAS, Vascular Dementia Assessment Scale; VMSB, Visual Memory Span Backward; VR, Visual Reproduction; WAIS-R, Wechsler Adult Intelligence Scale-Revised; WCST(−m), Wisconsin Card Sorting Test(−modified); WMS(−R), Wechsler Memory Scale(−Revised); WRAT-3, Wide-Range Achievement Test-Third Edition; 3MS, Modified Mini-Mental State Examination

**Abbreviations of neuroanatomical substrates:** AGVol, Angular Gyri Volume; ARWMCs, Age Related White Matter Changes; BA, Brain Atrophy; BVol, Brain Volume; B-to-ICVol, Brain-to-Intracranial Volume; CA, Cortical Atrophy; CCI, Cortical Cerebral Infarctions; CGMVol, Cortical Gray Matter Volume; CSFV, Cerebrospinal Fluid Volume; CVol, Cortical Volume; DFCVol, Dorsolateral Frontal Cortex Volume; DWMH, Deep White Matter Hyperintensities; DWML, Deep White Matter Lesions; ECVol, Entorhinal Cortex Volume; FA, Fractional Anisotropy; GMLV, (subcortical) Gray Matter Lesion Volume; GMVol, Gray Matter Volume; HC Vol, Hippocampal Volume; IBVol, Intracranial Brain Volume; LA, Leukoaraiosis; LAC, Volumes of Lacunes; LI, Lacunar Infarctions; MFCVol, Medial Frontal Cortex Volume; MFLVol, Medial Frontal Lobes Volume; MTA, Medial Temporal lobe Atrophy; NI, Number of Infarcts; NSSI, Number of Silent Small Infarcts; PMCVol, Posteromedial Cortex Volume; Pre. Vol, Precunei Volume; PVH, Periventricular Hyperintensities; PWML, Periventricular White Matter Lesions; RGMA, Regional Gray Matter Atrophy; SE, Sulcal Enlargement; SH, Subcortical Hyperintensities; SI, Small Infarcts; SRSI, Site of Recent Small Infarcts; TBVol, Total Brain Volume; TOFCVol, Total Orbital Frontal Cortex Volume; TWMVol, Total White Matter Volume; VaD, Vascular Dementia; VBR, Ventricle-to-Brain Ratio; VE, Ventricular Enlargement; Vol I, Volume of Infarcts; WBA, Whole Brain Area; WBVol, Whole Brain Volume; WMC, White Matter Change; WMH, White Matter Hyperintensities; WML, (subcortical) White Matter Lesions; WMLVol, (subcortical) White Matter Lesion Volume

## Studies that Found Unique Associations with Neuroanatomical Changes

Overall, of the 12 studies that examined the independent effects of WMC, four articles confirmed previous findings of significant associations between WMC and IADL. For example, Cahn et al. (1996) found that in patients with depression, subcortical hyperintensities accounted for an additional 18 % of the variance in IADL, in addition to age, depression severity, and neuropsychological test performance. Similar results have been reported in other populations such as patients with vascular dementia (Boyle et al. 2003b), AD patients with cerebrovascular disease (Chen et al. 2015), as well as in a longitudinal study in an elderly population (Inzitari et al. 2007).

Nine studies reported on the unique predictive value of hippocampal atrophy for IADL. Of these, four studies reported independent associations between hippocampal atrophy and IADL. Again, these findings pertain to a range of patient samples, including MCI and AD patients (Cahn-Weiner et al. 2007; Brown et al. 2011) as well as elderly people (Bennett et al. 2006; Verlinden et al. 2014).

Other studies reported unique associations with a neuroanatomical substrate that was reported in a single study only, such as orbitofrontal cortex volume (Taylor et al. 2003).

## Studies that Found Unique Neuropsychological Correlates

The findings with regard to the unique neuropsychological correlates of IADL are somewhat homogeneous. Of the nine studies that examined the independent effects of executive function, in addition to other functions, six reported unique associations with IADL. (i.e., Bennett et al. 2006; Boyle et al. 2003b; 2004; Cahn-Weiner et al. 2007; Christman et al. 2010; Mok et al. 2004). Furthermore, out of eight studies that examined the independent effects of memory function, in addition to other functions, five studies found unique associations with IADL (i.e., Bennett et al. 2002, 2006; Brown et al. 2011; Cahn-Weiner et al. 2007; Marshall et al. 2014). Some studies found effects of other measures, such as spatial span and arithmetic ability (Griffith et al. 2010) or of tests that tap psychomotor speed (Brown et al. 2011; Christman et al. 2010; Marshall et al. 2014) or attention (Bennett et al. 2002, 2006; Stoeckel et al. 2013).

Since executive functions are strongly related to WMC, it is crucial to determine whether the effects of executive function are independent of those from WMC; the same applies to memory and hippocampal atrophy. Six studies examined WMC together with executive control (as independent measure, not as part of one overall cognitive domain score) in relation to IADL. Of these, four studies noted independent effects of executive function only, one study found contributions of both executive function and WMC, whereas one study found no effects of these variables at all. In the same line, four studies examined memory and hippocampal measures concurrently, three of which reported independent effects of both of these predictors, whereas the fourth study reported no effects of these variables at all.

## Study that Reported Inconclusive Results

In a mixed group of healthy adults and early AD patients, Vidoni et al. (2010) demonstrated that impaired cognitive functioning was related to reduced gray matter volume in the medial frontal (AD patients) and in the middle frontal and precentral cortex (healthy group). According to the authors, this likely affects independent performance of IADL in part through a decline in associated executive aspects of functioning. The results suggest that cognitive function had both a strong direct effect on and mediated the influence of physical function on independence for those with AD. It is unclear, however, whether cognitive and neuroimaging correlates independently related to IADL.

## Effects of Clinical Condition

**Fig. 1:**
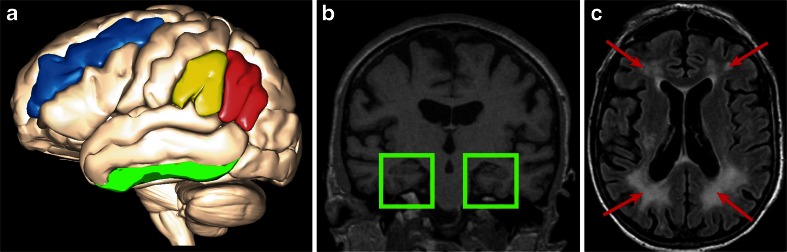
Overview of the main brain structures involved in IADL function; a) neocortical regions: middle frontal gyrus (blue) associated with IADL in non-demented older adults and AD patients, supramarginal gyrus (yellow) associated with IADL in AD, angular gyrus (red) associated in amnestic MCI and AD, inferior temporal gyrus (green) associated with IADL in AD; b) medial temporal lobe atrophy associated with impaired IADL function in non-demented older adults, MCI and AD (coronal T1-MRI showing the hippocampus and entorhinal cortex in green boxes); c) subcortical white matter lesions associated with IADL in vascular dementia, older adults with depressive symptoms and non-demented older adults (red arrows on transversal FLAIR-MRI indicating frontal and occipital white matter hyperintensities)

Of all studies included in the review, four reported on patient groups with a significant underlying cerebrovascular pathology, five on patients with Alzheimer pathology (amnestic MCI and/or AD, without including controls in the analysis), three on patients with depression (apart from controls), and six on elderly populations (without including patients in the analysis). In this section, we specifically focus on white matter and hippocampal measures, together with memory and executive function as neuropsychological domains, as these were the most important predictors of IADL overall. An overview of the most important MRI correlates of IADL is depicted in Fig. 1, specific disease states in which certain MRI variables are associated with IADL are reported in the caption.

In the studies that include patients with cerebrovascular pathology, three out of four found a unique effect of executive functioning on IADL, but only one out of four found a unique effect of underlying white matter pathology. One study found a unique effect of memory, but only two studies examined memory performance as an independent predictor in total; none of these studies examined hippocampal volume.

Of the five studies examining Alzheimer pathology, only one out of four studies that examined hippocampal volume found an independent effect on IADL. Two out of three studies found an effect of memory; two studies used executive function measures, but found no effect. None of these five studies measured white matter integrity.

None of the studies on clinical depression focused on independent measures of memory, executive function, or hippocampal volume. White matter integrity was examined in all three studies; only one reported an independent effect on IADL.

Finally, in six studies examining elderly populations, one out of four studies showed a unique effect of WMC whereas two out of three studies identified hippocampal volume as a unique correlate. Only two studies examined independent effects of memory and executive functioning, and only one reported that these cognitive functions independently relate to IADL.

## The Effect of Methodology

Consensus was reached between both raters and, overall, methodological quality of the studies was judged to be sufficient (see supplementary Online Recourse 1). One study did not reach the cut-off point of 5 (out of 9) that has been used to determine whether the methodological quality is sufficient (Stubbs et al. 2013). Six studies obtained a score of 5, five a score of 6, four a score of 7, and four a score of 8. Nonetheless, significant methodological differences exist across studies, which may account for the heterogeneous findings. In the next section, we will discuss the following potential confounders in relation to the study outcomes: how IADL was measured (self-report, informant-based or performance-based), how brain morphological changes were quantified (through visual or volumetric measures), and how the neuropsychological assessment was conducted (brief or detailed).

## The Effect of IADL Assessment

Of all studies included, six solely relied on self-report measures, nine used informant-based measures of IADL, two combined self-report with informant-based reports for their participants, two used performance-based indices of IADL, and one used informant-based IADL measures for their patients and self-report measures for their control group (see Table 1). The outcomes of the studies using self-report versus informant-based reports of IADL were comparable. In the seven studies that reported on correlates of self-report measures, five times (71.4 %) a unique effect of cognition was demonstrated, and four times (57.1 %) an independent effect of neuroimaging variables was reported. Of the 12 studies that reported correlates of informant-based IADL measures, these numbers were eight (66.7 %) and seven (58.3 %), respectively. At a more detailed level, no association was found between the type of IADL assessment and whether or not an effect of hippocampal atrophy, executive or memory deficits on IADL was found. The only exception was WMC, which were never related to IADL when self-report measures were used, whereas they were related to IADL in 50 % of the instances where studies relied on informant-reports. It should be noted, though, that informant-reports or performance-based indices of IADL were more likely employed in patients with pathological conditions such as stroke, MCI or dementia (83.3 % of the cases) compared to studies that solely focused on the elderly population, diabetic patients or depressed patients (54.5 % of all cases where cognitive and MRI correlates were considered).

## The Effect of MRI Assessment

Nearly all studies employed volumetric measurements to quantify morphological changes in the brain. Visual scoring, if used, was mainly employed to assess white matter integrity. Out of a total of 12 studies, five used visual and seven volumetric assessment of WMC. For both assessment methods, two studies reported unique effects of WMC on IADL, whereas five (volumetric) and three (visual) did not.

## The Effect of Neuropsychological Assessment

Several studies used a neuropsychological test battery to provide a detailed examination of cognitive functioning, tapping a wide array of functions such as memory, attention, executive functions, and psychomotor speed. Others, however, used a brief battery tapping some, but not all, of these functions, or even used a single broad cognitive screening instrument such as the MMSE. Further employment of cognitive outcome measures also differed between studies: some calculated a single cognitive outcome measure based on multiple neuropsychological tests, whereas others examined tasks/functions such as memory and executive function as independent constructs when examining associations with IADL.

To examine the effect of neuropsychological assessment, we made an distinction between: 1. Studies employing multiple neuropsychological tests tapping at least 3 distinct cognitive domains, 2. Studies conducting a brief neuropsychological examination, employing one or some neuropsychological tests and/or tapping less than 3 cognitive domains, and 3. Studies only using a screening instrument such as the MMSE. This resulted in six studies using a detailed and seven using a brief neuropsychological examination, and seven studies using a screening instrument. Of these studies, independent associations of cognition with IADL were noted for the majority of studies using detailed or brief neuropsychological examinations (both >65 %); for those studies using a screening instrument, only 42.9 % reported an independent association between this instrument and IADL.

## Discussion

The main goal of the present review was to examine to what extent cognitive and brain morphological changes independently predict IADL. The majority of studies discussed in the present review demonstrate that performance on neuropsychological tests for the domains memory (62.5 % of studies that examined this function) and executive functioning (66.7 % of studies) are both associated with present IADL impairment and predictive of future decline in IADL; overall, 83.3 % of all studies (that explicitly reported the effect of cognition) demonstrated a unique effect of cognition on IADL. Neuroimaging markers, independent of cognitive function, were also associated with IADL, but to a lesser extent. In total, 44.4 % of studies examining this brain structure reported that hippocampal volume was associated with impairment in IADL, and 33.3 % of studies found WMC predictive of IADL; overall, 60.0 % of the studies reported an independent effect of brain neuroanatomical changes. Whereas both these factors – cognitive dysfunction and brain morphological changes – are important and independent indicators of IADL, cognitive functioning appears to be a stronger predictor than morphological changes in the brain. Finally, whereas some unique effects of hippocampal atrophy were noted, that extended beyond the effects of episodic memory, WMC appear less predictive, particularly once executive functioning is simultaneously examined.

One possible explanation why episodic memory plays an important role in IADL is that our ability to fulfil future actions is strongly dependent on episodic memory processes (Einstein et al. 1992). Alternatively, a potential explanation is that episodic memory impairment/hippocampal atrophy merely reflects a disease severity effect in MCI and AD patients, in that more severe hippocampal atrophy/memory impairments and severe IADL impairments are both indicative of a more advanced disease state. Similarly, the association with hippocampal volume in healthy aging may reflect preclinical neuropathological changes as the result of neurodegenerative disease, such as AD (Apostolova et al. 2010; Den Heijer et al. 2006). Thus, the association between memory and hippocampal atrophy and IADL functioning may predominantly reflect an underlying neuropathological process rather than highlight a unique role of the hippocampus or memory function. Further studies that compare multiple, yet homogeneous, clinical populations that vary in the level of the extent to which memory and hippocampal volume are affected are needed in order to answer this question.

With respect to executive dysfunction, planning, sequencing capacity, and monitoring are prerequisites for successful IADL performance. In line, impaired executive processes may provide a possible explanation about how WMC may lead to impairment in IADL. That is, WMC may result in impaired executive function relevant for IADL due to disruption of frontal subcortical circuits (Cahn et al. 1996; Kiosses et al. 2000). However, we found that studies that concurrently examined the effects of WMC and executive functioning most consistently reported independent effects of executive function, but not WMC, on IADL. This suggests that the impairment in IADL associated with WMC is mediated by an impairment in executive function.

Although our review indicates that both neuropsychological and neuroanatomical changes may be important independent correlates of IADL, some limitations need to be addressed. Firstly, some studies reported a composite measure of cognitive functioning including several cognitive domains, making it impossible to detect the unique contribution of each domain in relation to IADL (Inzitari et al. 2007; Cahn et al. 1996; Vidoni et al. 2010). These studies therefore prohibit drawing firm conclusions regarding specific cognitive domains that are relevant for IADL.

Secondly, it is crucial to realize that the study outcome is largely determined by the variables that were included in the respective studies. For example, some studies only used the MMSE as the cognitive correlate (Steffens et al. 2002; Taylor et al. 2003; Hybels et al. 2014; Verlinden et al. 2014; Kochan et al. 2011; Chen et al. 2015), whereas others employed extensive neuropsychological test batteries (e.g., Cahn et al. 1996; Bennett et al. 2002; Christman et al. 2010). It is unclear whether MMSE scores (that are known to have little sensitivity for executive dysfunction or memory) will still be significantly associated with IADL once other functions, such as executive control, are considered as well. Patients with high education levels may also perform near ceiling level, which makes the MMSE inappropriate as a measure of cognitive functioning in highly-educated subjects (Franco-Marina et al. 2010). These shortcomings are substantiated by our examination of the outcomes in relation to the neuropsychological examination; unique associations with IADL were less often reported when cognitive screening instruments were used, compared to studies that employed more detailed neuropsychological examinations.

Similarly, with respect to potential morphological correlates, a significant portion of studies focused solely on WMC in relation to IADL (Boyle et al. 2004; Hybels et al. 2014; Pantoni et al. 2006; Inzitari et al. 2007). Likewise, the unique contribution of variables that are strongly interrelated and also predict IADL, such as executive function and WMC, or hippocampal atrophy and memory, needs specification in future studies. Most studies found either an independent effect of morphological changes or an independent effect of neuropsychological correlates. Only a small number of studies found concurrent independent effects of both potential correlates, and only few identified independent contributions of interrelated predictors, such as concurrent independent effects of WMC and executive function.

Even though we found evidence that executive function problems, and at one occasion WMC, predicted IADL in patients with vascular pathology, hippocampal pathology was not examined in these patients. However, hippocampal atrophy is a common observation in patients with vascular cognitive impairment (Liu et al. 2014, although the atrophy is potentially less severe than in AD, see Kim et al. 2015) and is therefore likely to predict IADL in this population as well. In turn, none of the studies in patients with Alzheimer pathology examined whether WMC played an important role, despite the fact that WMC are also commonly observed in AD patients (Burton et al. 2006; Kandiah et al. 2015). Related to this, although true neurocognitive deficits are likely present in neurodegenerative disorders such as AD, factors such a lack of motivation may explain such deficits in depressed patients (Kiosses and Alexopoulos 2005). Whether this holds true for the current findings is unclear, as two studies have demonstrated independent effects of brain morphological changes, namely of white matter pathology (Cahn et al. 1996) and orbitofrontal cortex volume (Taylor et al. 2003), on IADL. This suggests that much remains to be done in identifying those variables that most strongly predict IADL in different clinical populations.

One of the reasons for the mixed results may relate to the method of reporting on IADL questionnaires. For example, Tan et al. (2009) found that executive function was more strongly associated with informant-reports than self-reports in MCI. In addition, informant-reports of IADL were able to accurately distinguish levels of functional independence, whereas self-report measurements could not (Farias et al. 2005). In particular, when IADL variables are obtained from self-reports in depressed individuals, results may not represent true limitations, as a depressed mood may influence the perception of functional impairment. Furthermore, most of the studies reviewed used questionnaire-based assessment and only two studies used performance-based assessments. Questionnaire-based and performance-based assessment of IADL may call upon different executive problems or processes (Cahn-Weiner et al. 2002). Consequently, cognitive predictors may vary as a result of the type of IADL assessment protocol.

In addition, it is possible that the type or format of the IADL questionnaire used may influence the cognitive variables which are necessary to perform the task. Although nine studies used the same IADL subscale of Lawton and Brody, with which the remaining questionnaires show overlap, there are also differences in the type of IADL items being assessed. For instance, some of the questionnaires include items such as being able to talk about current events, a tendency to dwell in the past, or paying attention and understanding while reading or watching television. Moreover, a variation is present in the way questions about IADL items are formulated. Whereas one questionnaire may inquire more superficially about performing financial administration, the other will examine the same task more thoroughly. The interpretation of the relation found between cognitive variables and IADL is further complicated by a variety in formats of items to be rated. The scales differ in their range of rating points between dependence and independence in a task, which make them more or less sensitive to detect functional decline. In sum, a number of factors related to the questionnaires reviewed influence the variance in prediction of IADL performance.

Some discussion about the methodological quality of the included studies in this review is in place. The methodological quality of the studies included was rated using the Newcastle-Ottawa Quality Assessment Scale. Overall, the quality of nineteen studies was judged to be sufficient; only one study did not reach the cut-off point of 5. Nevertheless, the quality of the studies reviewed could certainly be improved given the fact that eleven studies achieved just a score of 5 or 6. Moreover, the possibility of a publication bias cannot be ruled out. In order to take into account the effect of a publication bias, the majority of the key authors of the studies included were contacted to inquire about the existence of unpublished study results on this topic. Only three authors responded to our request; none of these authors were aware of unpublished studies. Furthermore, considering the small number of included studies, we could not be too strict regarding the inclusion versus exclusion criteria, for example by including only those studies with a comprehensive neuropsychological examination.

Clearly, research examining the combined impact of cognitive and neuropathological factors on instrumental activities of daily living is still in its infancy. Additional research is needed to clarify the predictors of functional impairment in elderly people and to establish the relative contribution of specific neuropsychological and morphological deficits. Given the impact of pre-frontal dysfunction and hippocampal atrophy on IADL, thorough evaluations of executive and memory dysfunction in patients seen in memory clinics, combined with neuroimaging, may assist in identifying individuals at risk for functional disability and provide important information regarding treatment planning.

To summarize, the present review provides clear evidence that executive function and memory, together with hippocampal atrophy – and to a lesser extent white matter changes – play crucial roles in maintaining an independent lifestyle. Studies systematically examining these neuropsychological and morphological factors concurrently in different patient populations are needed to identify the uniqueness of these potential correlates for IADL. In addition, longitudinal studies are needed to determine whether these cognitive and morphological deficits are actually causally related to disability. If so, interventions to treat cerebrovascular disease and to prevent its worsening may have a significant impact on functional status.

## Electronic supplementary material

Supplementary Online Recourse 1 (DOCX 16 kb)
