# Supplementary material for: The Combined Effect of Neuropsychological and Neuropathological Deficits on Instrumental Activities of Daily Living in Older Adults: a Systematic Review
Source: Neuropsychol Rev. 2016 Jan 5;26:92–106. doi: 10.1007/s11065-015-9312-y (PMC4762929; doi:10.1007/s11065-015-9312-y)
Supplement: Supplementary file 1 — Supplementary Online Recourse 1 (DOCX 16 kb) [file 11065_2015_9312_MOESM1_ESM.docx]

Supplementary Table

Newcastle-Ottawa Quality Assessment Scale

| Cross sectional studies | Selection | Comparability | Outcome |
| --- | --- | --- | --- |
| Bennett et al., 2006  Boyle et al., 2003  Brown et al., 2011  Cahn et al., 1996  Christman et al., 2010  Griffith et al, 2010  Hybels et al., 2014  Mok et al., 2004  Steffens et al., 2002  Stoeckel et al., 2013  Taylor et al., 2003  Vidoni et al., 2010 | ***  *  **  *  **  *  *  ***  *  *  *  ** | **  *  **  *  **  **  **  **  **  **  ** | ***  ***  ***  ***  **  ***  **  **  **  ***  **  *** |
| Cohort studies | Selection | Comparability | Outcome |
| Bennett et al., 2002  Boyle et al., 2004  Cahn-Weiner et al., 2007  Chen et al., 2014  Inzitari et al., 2007  Kochan et al., 2011  Marshall et al., 2014  Verlinden et al., 2014 | ***  *  ***  ***  ****  *  **  ** | **  **  **  *  *  **  **  ** | ***  **  ***  **  ***  ***  **  *** |
|  |  |  |  |

The combined effect of neuropsychological and neuropathological deficits on instrumental activities of daily living in older adults: a systematic review, Neuropsychology Review.

Eduard J. Overdorp ^1^, Roy P. C. Kessels ^2,3,4^, Jurgen A. Claassen ^2,4^, Joukje M. Oosterman ^2^.

^1^ Gelre Medical Centre, Department of Medical Psychology, Zutphen, The Netherlands.

^2^ Radboud University, Donders Institute for Brain, Cognition and Behaviour, Nijmegen, The Netherlands.

^3^ Radboud university medical center, Department of Medical Psychology, Nijmegen, The Netherlands.

^4^ Radboud university medical center, Department of Geriatric Medicine and Radboud Alzheimer Center, Nijmegen, The Netherlands. Corresponding author: Joukje Oosterman, email: [j.oosterman@donders.ru.nl](mailto:j.oosterman@donders.ru.nl)
